# Deciphering the Role of Ca^2+^ Signalling in Cancer Metastasis: From the Bench to the Bedside

**DOI:** 10.3390/cancers13020179

**Published:** 2021-01-07

**Authors:** Abeer Alharbi, Yuxuan Zhang, John Parrington

**Affiliations:** 1 Department of Pharmacology, University of Oxford, Oxford OX1 3QT, UK; yuxuan.zhang@pharm.ox.ac.uk; 2 Pharmaceutical Sciences Department, College of Pharmacy, King Saud Bin Abdul-Aziz University for Health Sciences, Riyadh 11426, Saudi Arabia

**Keywords:** calcium, Ca^2+^ signals, metastasis, cancer

## Abstract

**Simple Summary:**

Ca^2+^ dyshomeostasis is implicated in several key pathophysiological processes attributed to cancer metastasis biology. Here, we decipher the role of intracellular and extracellular Ca^2+^ signalling pathways in processes that contribute to metastasis at the local level (involving cell proliferation, adhesion, motility, invasion, migration and the epithelial-mesenchymal transition) and also their effects on cancer metastasis globally. Ca^2+^ proteins are potential candidates for cancer biomarkers and druggable targets for future metastatic cancer therapy.

**Abstract:**

Metastatic cancer is one of the major causes of cancer-related mortalities. Metastasis is a complex, multi-process phenomenon, and a hallmark of cancer. Calcium (Ca^2+^) is a ubiquitous secondary messenger, and it has become evident that Ca^2+^ signalling plays a vital role in cancer. Ca^2+^ homeostasis is dysregulated in physiological processes related to tumour metastasis and progression—including cellular adhesion, epithelial–mesenchymal transition, cell migration, motility, and invasion. In this review, we looked at the role of intracellular and extracellular Ca^2+^ signalling pathways in processes that contribute to metastasis at the local level and also their effects on cancer metastasis globally, as well as at underlying molecular mechanisms and clinical applications. Spatiotemporal Ca^2+^ homeostasis, in terms of oscillations or waves, is crucial for hindering tumour progression and metastasis. They are a limited number of clinical trials investigating treating patients with advanced stages of various cancer types. Ca^2+^ signalling may serve as a novel hallmark of cancer due to the versatility of Ca^2+^ signals in cells, which suggests that the modulation of specific upstream/downstream targets may be a therapeutic approach to treat cancer, particularly in patients with metastatic cancers.

## 1. Introduction

Cancer is a serious public health condition globally. Metastasis is a significant hallmark of cancer, defined as the transition of cancer cells from their original site to another site, and accounts for ~90% of cancer-related mortalities [1]. Metastasis is a complex phenomenon that involves multiple phases (from the translocation from the primary site to the colonization of the secondary site) and several pathophysiological processes (including cell proliferation, adhesion and motility; tumour invasion and migration; angiogenesis; and the epithelial-mesenchymal transition) which interact with each other at a local level to develop metastatic cancer at a global level. It is a fundamental phenomenon in our understanding of the underlying molecular mechanisms related to cancer pathogenesis; hence, it is a viable target for cancer therapy and approaches to prevent and target metastatic cancer have drawn scientific attention for several decades and remain of great interest in decoding cancer biology. Ca^2+^ is a versatile second messenger, and its homeostasis is critical to hindering the development of metastatic cancer at both the intracellular and extracellular levels. Intracellular and extracellular Ca^2+^ signalling is implicated in several key pathophysiological processes which are attributed to tumour metastasis and progression [2,3,4,5,6,7,8,9].

**Figure 1:**
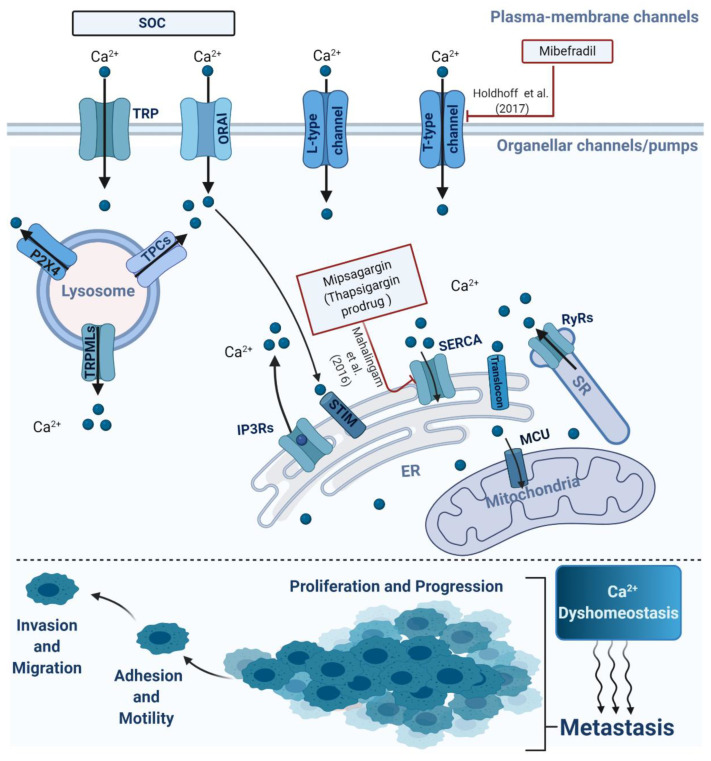
Schematic representation of the main intracellular or extracellular calcium channels involved in metastasis. The alterations of Ca^2+^ homeostasis via organellar or plasma channels/pumps were implicated in several processes attributed to cancer metastasis, involving cell proliferation, invasion, migration and progression.

Importantly, dysregulation of spatiotemporal Ca^2+^ homeostasis at both intracellular and extracellular levels, in terms of spatiotemporal oscillations or waves, alters cellular physiological processes at the local level leading to metastatic cancer globally (shown in Figure 1). There are two main Ca^2+^ signalling pathways: intracellular (local) and extracellular (global). The implications of their communication and complementary interplay for the development of metastatic cancer are becoming extremely difficult to ignore. It has become evident that intracellular calcium channels, including inositol 1,4,5-trisphosphate (IP_3_) receptors (IP_3_Rs), transient receptor potential cation channels (TRPML, mucolipins), and two-pore channels (TPCs), play roles in the modulation of key processes that regulate tumour progression and migration [9,10,11,12]. Our recent review discussed briefly the role of two-pore channel 2 (TPC2) in tumour cell migration [9]. In addition, extracellular Ca^2+^ signalling pathways, via calcium-sensing receptor (CaSR)and store-operated calcium entry (SOCE), have been shown to contribute to pathophysiological processes that promote metastasis [13,14]. A growing quantity of experimental evidence and a limited number of clinical trials suggest a potential clinical application of Ca^2+^ modulators and their upstream/downstream targets as a therapeutic approach to treat metastatic cancer. Recently, a considerable amount of literature has been produced around the theme of Ca^2+^ signalling in cancer, particularly its pivotal role in pathophysiological processes towards cancer metastasis. Here, we look at the role of Ca^2+^ signalling at both the intracellular and extracellular levels in cancer metastasis, which will contribute to a deeper understanding of cancer pathogenesis and permit us to further investigate Ca^2+^ signalling as a regulator of tumour progression and metastasis.

## 2. Intracellular Calcium Signalling in Metastasis

### 2.1. Endoplasmic and Sarcoplasmic Reticulum Ca^2+^ Channels/Pumps

Endoplasmic and sarcoplasmic reticulum Ca^2+^ channels/pumps include inositol 1,4,5-trisphosphate (IP_3_) receptors (IP_3_Rs), ryanodine receptors (RyRs), the translocons, and sarco-endoplasmic reticulum Ca^2+^ reuptake pump (SERCA). SERCA acts as a mobiliser of Ca^2+^ from the cytosol into the ER to maintain cytoplasmic Ca^2+^ homeostasis. It consists of three major isoforms (SERCA1-3) [15]. Chung et al. (2006) found that high SERCA2 expression was correlated with lymph node metastasis, advanced stages of tumourigenesis, and significantly shorter survival compared to low SERCA2 expression in patients with colorectal cancer [16]. Unlike earlier findings, high SERCA3 expression was significantly associated with longer survival, negatively correlated tumour node metastasis (TNM) staging and distant metastases, but not with lymph node metastasis in patients with gastric carcinomas [17]. Shi et al. (2018) showed that SERCA is involved in Yap (Yes-activated protein)-mediated hepatocellular carcinoma metastasis [18].

The emerging role of intracellular Ca^2+^ signalling in cancer cell migration is not a recent discovery. Rondé et al. highlighted the intracellular Ca^2+^ oscillations which are linked to cell migration in U-87MG cells (an in vitro model of malignant glioma) via IP_3_Rs, but not ryanodine receptors [19]. A previous study found that ryanodine receptor isoform-2 (RYR2) gene expression was upregulated by 45-fold in epidermal growth factor (EGF)-treated MDA-MB-468 cells (mesenchymal-like state) compared to MDA-MB-468 cells (epithelial-like state), suggesting that the involvement of the RYR2/Ca^2+^ signalling pathway in the EGF-induced epithelial-mesenchymal transition (EMT) in breast cancer, which is a critical process for cell adhesion, invasion and migration, ultimately leads to a metastatic state [20]. Recently, Fukushima et al. have uncovered the role of translocation associated membrane protein 2 (TRAM2), a component of the translocon, in metastasis [21]. They have shown that TRAM2 knockdown eliminated metastatic traits—including cell invasion and transendothelial migration in oral squamous cell carcinoma (OSCC) cells—by modulating the expression of matrix metalloproteinases. Their study found that Ca^2+^ permeability via translocon mediates cancer progression [21]. Ca^2+^ release in the intracellular compartment is mainly mediated by IP_3_Rs, which are located on the ER [16]. There are three isoforms: IP_3_R type 1 (IP_3_R1), IP_3_R type 2 (IP_3_R2), and IP_3_R type 3 (IP_3_R3) [18]. The release of Ca^2+^ from the ER to the cytosol via IP_3_Rs is mainly trigged by IP_3_ and Ca^2+^ [22]. Whole-exome sequencing (WES) conducted by Hedberg et al. in patients with head and neck squamous cell carcinoma (HNSCC) underpinned the potential clinical utility of IP_3_R3 as a prognostic biomarker. They discovered genetic mutations in IP_3_R3 in metastatic or recurrent HNSCC cancers, but not in the primary tumour [23]. IP_3_R3 overexpression is implicated in various types of cancer including breast, colorectal, cholangiocarcinoma, gastric and glioblastoma, and promotes cancer progression by enhancing metastatic phenotypes [24,25,26,27,28]. When siRNA was used to silence IP_3_R3 in an in vitro model of breast cancer, this was shown to attenuate cell migrations induced by Ca^2+^ oscillations [24]. Recent data showed that IP_3_R3 function was drastically impaired by epidermal growth factor receptor (EGFR) and tyrosine-protein kinase (MET) inhibitors in oncogene-driven non-small cell lung cancer (NSCLC), thus raising intriguing questions regarding the possibility of targeting upstream or downstream regulator or effector proteins of IP_3_R3 to treat metastatic cancer patients, particularly those with NSCLC [29].

In contrast to the findings which demonstrated that the IP_3_R3/Ca^2+^ signalling pathway is critical for cancer invasion and migration in vitro, IP_3_R2 was found to be a key mediator of ER Ca^2+^ signals which mediate migration in human lung cancer cells (A549 cell line) [30].

Taken together, these findings emphasise the critical role of Ca^2+^ signalling from the ER, mainly via IP_3_Rs, which acts as a key regulator of several pathophysiological processes related to tumour progression and migration. Despite substantial in vitro evidence that has led to the recognition of emerging roles of IP_3_Rs as modulators of Ca^2+^ signalling and enhanced metastatic traits, further studies utilizing in vivo IP_3_R knockout mouse models will help to further reveal the molecular mechanisms of IP_3_Rs as mediators of metastasis.

### 2.2. Endolysosomal Ca^2+^ Channels

TPCs, TRPML, and P2X(4) receptors are intracellular Ca^2+^ permeable channels and are located in the endolysosomal compartment, which consists of early, late, and recycling endosomes, lysosomes, and autophagosomes. While they have an evident role in the involvement of endolysosomal Ca^2+^ signalling pathways in cancer phenotypes from tumour initiation to cancer cell migration [11], the molecular mechanisms underlying endolysosomal Ca^2+^ signal-mediated metastasis remains speculative. Two-pore channel type 1 (TPC1) and two-pore channel type 2 (TPC2) are two isoforms of the two-pore channel superfamily, expressed in mammalian cells. Recently, the effects of TPCs and particularly TPC2 on pathophysiological processes related to metastatic cancer have been observed in in vitro and in vivo cancer models [9]. TPC1- or TPC2-deficient T24 cells (an in vitro model of bladder cancer) generated by siRNA showed a significant decrease in metastatic phenotypes cell adhesion and migration compared to control cells [31]. In the same study, diminished TPC function achieved either by silencing using siRNA or pharmacological inhibition by Ned-19 or tetrandrine in T24 cells was shown to alter β1-integrin recycling, which is involved in cell motility and invasion. This ultimately hinders tumour metastasis [31]. Notably, the inhibition of TPC2 function using siRNA or inhibitors in an in vivo mouse mammary cancer model has been shown to significantly reduce the formation of lung metastasis [31]. These results differ from recent evidence demonstrating that the downregulation of TPC2 expression or TPC2 knockout promotes tumour metastasis in melanoma cells generated from an advanced stage of tumourigenesis [32]. The controversy about whether TPC2/Ca^2+^signaling in metastatic cancer promotes or hampers metastatic traits—such as tumour cell adhesion, motility, invasion and progression—might reflect TPC2 having differential roles in different types or stages of cancer. Three isoforms of transient receptor potential cation channels (TRPMLs) found in mammals are TRPML1, TRPML2, and TRPML3 [33]. TRPML1 knockdown conducted with siRNA in HepG2 cells (an in vitro human hepatocellular liver carcinoma model) impaired invasion and attenuated cell migration compared to WT HepG2 cells [34]. Additionally, this study identified for the first time the mechanism of action of tetrabromobisphenol A (TBBPA), a toxin that has been linked to hepatic cancer invasion and migration, finding that TBBPA evoked endolysosomal Ca^2+^ signals upon binding to TRPML1 [34]. An increased expression of transient receptor potential mucolipin1 (TRPML1) was also detected in advanced stages (III–IV) compared to early stages (I–II) of tumourigenesis in patients with non-small-cell lung cancer (NSCLC); TRPML1 silencing or inhibition in vitro impaired pathophysiological processes related to metastatic NSCLC cancer, indicating that enhanced expression of mucolipin 1 was involved in cancer progression and metastasis by promoting cell invasion, proliferation and migration in NSCLC [35]. TRPML-2 mRNA and protein levels were found to be elevated in brain cancer patients and correlated with advanced pathological grades (from astrocytoma (I) to glioblastoma (IV)) [36]. TRPML-2-deficient U251 and T98 cells (an in vitro model of glioblastoma) showed a reduction in cell proliferation involving the inhibition of AKT and ERK1/2 signalling [36], suggesting that TRPML-2 acts as a regulator of ERK1/2 and AKT signalling pathways in glioblastoma cell proliferation.

Recently, TRPML3 was discovered to be one of the nine gene signatures predicting overall survival in patients with pancreatic cancer [37]. Downregulation of TRPML3 expression acts as a protective factor in the prognostic nomogram established for pancreatic cancer [37]. The above findings suggest the possibility of the clinical utility of TRPML subtypes as a potential distinct prognostic marker for cancer progression and overall survival in various cancer subtypes. The P2X(4) receptor is expressed in the endolysosomal system and modulated by ATP and pH [38]. To our knowledge, no previous study has investigated the role of P2X(4) receptors in metastatic traits. Endolysosomal Ca^2+^ signals have attracted growing interest as a novel biomarkers or therapeutic targets for metastatic carcinoma. Further studies to confirm these findings through in vivo mouse models or a prospective large cohort of cancer patients are required.

Despite the substantial literature that implicates the different roles of lysosomal Ca^2+^ release channels in cancer metastasis, there is a lack of evidence for how these lysosomal Ca^2+^ channels may interact to mediate development of metastatic cancer at a global level. We speculate that lysosomal Ca^2+^ dyshomeostasis contributes to metastatic phenotypes with distinctive roles for these channels and possible crosstalk that requires further investigation to expand our knowledge of the pathophysiology of cancer metastasis biology. The mobilisation of cytosolic Ca^2+^ into endolysosomal compartments is poorly understood and remains enigmatic. Garrity et al. found that the ER plays a role in the Ca^2+^ refilling of lysosomes [39], and we infer that it occurs via an unidentified Ca^2+^ transporter.

### 2.3. Intracellular Ca^2+^ Signalling and Ca^2+^-Activated K^+^ Channels (K_Ca_) in Metastasis

Intracellular calcium oscillations activate Ca^2+^-activated K^+^ channels, involving intermediate (K_Ca3.1_) and large conductance (K_Ca1.1_), were found to promote tumour cell proliferation, migration and progression [40,41,42,43]. K_Ca3.1_ and K_Ca1.1_ differ in their Ca^2+^ sensitivities. K_Ca3.1_ requires a small physiological alteration in Ca^2+^, while K_Ca1.1_ responds to a large change in Ca^2+^ [44]. Several studies have provided substantial evidence that K_Ca3.1_ and K_Ca1.1_ contribute to glioblastoma metastasis biology [45,46,47,48]. Growing evidence is linking K_Ca3.1_ to glioma cell invasion and migration [46,49,50], and recent data has implicated that K_Ca3.1_ is upregulated in high-radiation dose-induced glioblastoma cell invasion [51]. K_Ca1.1_ was shown also to play a role in radiation-enhanced glioblastoma migration in in vitro and in vivo murine models [52]. Pharmacological inhibition of K_Ca1.1_ diminished migratory capability of glioblastoma cells induced by hypoxia in U87-MG cells [47]. Overall, these findings indicate the indirect involvement of intracellular Ca^2+^ signalling-mediated cell invasion and migration via either K_Ca3.1_ or K_Ca1.1_ in glioblastoma. Further work is required to underscore the crosstalk between these channels and intracellular Ca^2+^ signalling at the molecular level to understand the pathophysiology behind the roles of these channels in glioblastoma metastasis biology. These channels might represent viable clinical tools that can enhance the efficiency of detection and guide the treatment of glioblastoma patients.

## 3. Extracellular Components of Ca^2+^ Signalling in Metastasis

Apart from providing structural supports for cells to form organs and tissues, the extracellular matrix (ECM) and extracellular proteins play other vital roles in various cell functions. Proteins in the extracellular space and on the cell membranes form a complicated network which initiates signalling cascades in the intracellular space; such signalling cascades regulate multiple aspects of cell behaviour including determination, differentiation, proliferation, and migration [53]. Although extracellular proteins have been less studied in relation to cell signalling than intracellular components, abundant evidence of their critical functions has been revealed in the past decade. Here we review some extracellular proteins related to Ca^2+^ signalling with particular emphasis on their mechanisms of action and functional roles in processes linked to cancer, especially metastasis.

### 3.1. Calcium-Sensing Receptor (CaSR)

As the ECM is the largest Ca^2+^ reservoir in multicellular organisms, macromolecules in the extracellular space directly bind to receptors on the cell surface resulting in Ca^2+^ entering the cell [54]. One such receptor is the calcium-sensing receptor (CaSR), a ubiquitously expressed G protein-coupled receptor sensing extracellular Ca^2+^ levels and controlling Ca^2+^ homeostasis by regulating parathyroid hormone release in the parathyroid gland and inhibiting Ca^2+^ reabsorption in the kidney [55,56]. The functions of the CaSR in the parathyroid gland and kidney have long been well recognized but a recent study reported that the CaSR has played pivotal roles in diverse processes such as inflammation, apoptosis, migration and proliferation. In particular, its paradoxical role in cancer has aroused a lot of interest [57]. The CaSR suppresses cell proliferation and induces terminal differentiation in parathyroid and colon tumors, as shown by recent studies which provided abundant evidence that overexpressing the CaSR suppressed the proliferation of colorectal cancer cell both in vivo and in vitro [58,59], while inversely, it acts as an oncogene in prostate, testicular, ovarian, and breast cancer, especially bone metastasis in breast and prostate cancer [60,61]. As early as 2006, Liao et al. demonstrated that elevated extracellular Ca^2+^ facilitated skeletal metastasis of prostate cell lines and that this effect was associated with an up-regulated CaSR which mediated the influx of extracellular Ca^2+^ triggering the AKT signalling pathway, but extracellular Ca^2+^ influx had no effect in prostate cancer cells derived from a lymph node metastasis [57]. Around the same time, Mihai et al. provided clinical evidence that CaSR-positive tumors were more likely to develop bone metastasis in breast cancer, by assessing the intensity of CaSR expression in the primary tumor histological sections [62]. This effect was later shown to have involved extracellular-signal-regulated kinase (ERK1/2) and phospholipase C beta (PLCβ) as downstream effectors [63]. Using similar methods as Mihai et al., Feng et al. identified a promotion function for the CaSR in metastatic prostate cancer; thus by pathological and statistical analysis, they found that compared to non-metastatic prostate cancer tissue, metastatic cancer tissue specifically expressed a higher level of the CaSR [61]. In 2014, Joeckel et al. demonstrated in renal cell carcinoma (RCC) cells that the CaSR mediated the promotion function of extracellular Ca^2+^ on tumor cell proliferation and bone metastasis via activation of the PI3K (phosphatidyl-inositol 3-kinase)/AKT pathway, the PLCγ-1 pathway, and the mitogen activated protein kinase (MAPK) cascades [64,65].

Taken together, the findings show that binding of these proteins to the CaSR initiates intracellular Ca^2+^ signaling cascades which lead specifically to the bone metastasis of multiple cancers, indicating that the CaSR can be a treatment target and also a diagnostic indicator of metastasis to bone.

### 3.2. Store-Operated Calcium Entry (SOCE)

One of the major mechanisms that regulate and remodel Ca^2+^ influx pathways in tumour progression is store-operated calcium entry (SOCE), the process in which Ca^2+^ passes through the cell membrane upon the depletion of intracellular Ca^2+^ stored in the endoplasmic reticulum (ER) [66,67]. Growing evidence has shown that SOCE and its molecular determinants are involved in various cell behaviours including proliferation, angiogenesis, invasion, and migration in some types of cancers [68,69,70].

#### 3.2.1. ORAI

As an important determinant of SOCE, ORAI proteins, which form a store-operated calcium selective ion channel, have been linked to roles in the development of cancer cells. ORAI forms calcium release-activated channels (CRAC) on the cell surface and interacts with stromal interaction molecule 1 (STIM1) which senses the Ca^2+^ concentration inside the ER and regulates SOCE [71]. In 2014, Umemura et al. reported that melanoma cell proliferation and metastasis were significantly suppressed by either genetically down-regulating ORAI or pharmacologically inhibiting SOCE [68], and since it has long been recognized that in melanoma cells, proliferation is regulated via ERK signalling, and migration is regulated via calpain-dependent actin dynamics [72], Umemura et al. proved that both these regulatory mechanisms were initiated by SOCE [68]. In hepatocarcinoma tissues, Tang et al. reported that genetic downregulation of ORAI1 or pharmacological inhibition of SOCE using SKF96365 improves 5-FU-induced autophagy and cell death in HepG2 cells (an in vitro model of hepatocarcinoma) [73]. ORAI mediated SOCE also leads to metastasis in acute myeloid leukemia, as reported by Diez-Bello et al. Genetic knockdown of ORAI1 and ORAI2 in the promyeloblastic cell line HL60, attenuated cell proliferation and metastasis via promotion of the phosphorylation of the focal adhesion kinase (FAK), which was shown to be essential for cell migration and invasion [66,74]. The link between FAK and another ORAI isoform, ORAI3, and their roles in tumorigenesis, was also reported by Motiani et al. in breast cancer cells [75]. Of all the ORAI isoforms, ORAI1 is the most ubiquitously expressed and the most well studied, however, future studies may focus on determining whether different ORAI isoforms have varying roles in different cancer types or at different stages of tumourigenesis.

#### 3.2.2. Stromal-Interaction Molecule (STIM)

Stromal-interaction molecule (STIM) is a Ca^2+^ sensor in the ER that triggers SOCE activation. How STIM regulates cancer progress is controversial. Chen et al. revealed, through in vitro studies, mouse models, and clinical analyses, that STIM1-dependent signalling regulates proliferation, migration, and angiogenesis in cervical cancer cells [76]. STIM1 also affects invasion and migration of gastric cancer cells, possibly through an unknown pathway independent of the MEK/ERK signaling, as reported by Xu et al. [77].

#### 3.2.3. TRP Channels

Alterations of Ca^2+^ homeostasis via transient receptor potential (TRP) channels were implicated in several processes attributed to cancer metastasis, practically cell proliferation and migration, which are two of cancer’s hallmarks. TRP is a superfamily of cation channels localised in the plasma membrane and composed of subfamilies, such as transient receptor potential canonical (TRPC), transient receptor potential vanilloid (TPRPV) and transient receptor potential melastatin (TRPM) [78]. Although previous studies have provided evidence of the involvement of various isoforms of TRPC, such as TRPC1, TRPC4, TRPC5 and TRPC6, in regulating pathophysiological processes related to tumour metastasis [79,80,81,82,83], and several reviews [84,85,86,87] have also discussed it, current studies focus mainly on the role of TRPC6/Ca^2+^ signalling in cancer metastasis at the global level in various types of cancers and revealed the emerging roles of TRPC3 in melanoma metastasis at the local level. Oda et al. (2017) found that TRPC3 acts as a modulator of melanoma cell proliferation and migration in in vitro and in vivo models (using the C8161 human melanoma cell line) in a mechanism involving (matrix metallopeptidase 9) MMP9 activation [88]. Inhibition of TRPC6/Ca^2+^ signalling either pharmacologically (using SKF-96365) or by genetic downregulation using siRNA showed a significant reduction in A549 cell (an in vitro model of NSCLC) proliferation by arresting the cell cycle at the S-G2/M phase and invasion [89]. Therefore, inhibiting the effects of TRPC6/Ca^2+^ signalling may serve as a viable therapeutic target for patients with NSCLC metastatic cancer, and it warrants further investigation in an in vivo model. Recently, the novel roles of the Na^+^/Ca^2+^ exchanger 1 (NCX1) and TRPC6 were deciphered in modulating transforming growth factor-beta (TGFβ), which plays a vital role in various aspects of human hepatocellular carcinoma metastasis, involving hepatic cell invasion and migration [90]. Recent evidence has shown that Ca^2+^ signalling via TRPC6 acts as a regulator of *Helicobacter pylori*-mediated gastric cancer invasion and migration involving activation of the Wnt/β-catenin signalling pathway in AGS and MKN45 cells [91]. A growing body of evidence highlights the contribution of various TRPM isoforms, including TRPM2, TRPM4, TRPM5, TRPM7 and TRPM8, in cancer metastasis biology [92,93,94,95,96,97,98,99,100]. Recent scientific attention was given to TRPM8 in bladder cancer metastasis. Wang et al. demonstrated that TRPM8 modulates cell proliferation and migration, ultimately leading to the development of bladder cancer metastatic phenotypes [101]. Knockdown of TRPM8 attenuates bladder cancer proliferation and progression in T24 cells and slows down tumour growth and progression in a murine model of human urinary bladder cancer [101]. The availability of a TRPM8 antagonist (PF-05105679), which has been tested in humans (phase 1 trial, NCT01393652) [102], raises a translational question regarding the possibility of modulating TRPM8 as a therapeutic approach and giving it as adjuvant therapy for patients with metastatic cancer after adequate data for its safety and tolerability (I.e. through clinical validation) have been obtained and an analogue to overcome one potential therapeutic limitation (causing a hot feeling in patients) has been developed that might greatly help the development of an anti-neoplastic agent to treat metastatic cancer.TRPV1, TRPV2 and TRPV4 are reported to regulate pathophysiological processes related to metastatic traits [103,104,105,106,107]. Recently, growing evidence has shown that TRPV4 modulates epithelial-mesenchymal transition and cytoskeleton promoting cancer metastasis [108,109]. TRPV4/Ca^2+^ signalling enhances gastric cancer progression in an in vitro model of gastric cancer (HGC-27 and MGC-803 cells) and is significantly correlated with aggressive features (involving depth of tumour invasion and lymph node metastasis) in gastric cancer patients, which suggests its clinical utility as a biomarker to predict the prognosis in patients with gastric cancer [108]. Li et al. underpinned the role of TRPV4/Ca^2+^ signalling-promoted endometrial cancer metastasis through the modulation of the cytoskeleton in a mechanism involving the activation of the RhoA (Ras homolog gene family member A)/ROCK1(Rho-associated protein kinase 1) signalling pathway [109]. Further studies are required to expand our cancer biology knowledge of the molecular mechanisms underlying the TRP modulation of metastasis and the identification of novel targets/biomarkers to treat metastatic cancer.

#### 3.2.4. Mitochondrial Ca^2+^ Uniporter and SOCE Crosstalk

The mitochondrial Ca^2+^ uniporter (MCU) mobilizes mitochondrial Ca^2+^ signalling from the cytosol into mitochondria. The cellular mechanisms underlying the regulation of Ca^2+^ signalling via MCU in pathophysiological processes that are related to metastatic cancer [110,111] and its links to store-operated Ca^2+^ entry-mediated tumour metastasis have been investigated [112]. Several studies have shown that MCU plays a pivotal role in breast cancer progression and metastasis and that it is a candidate therapeutic target and biomarker for breast cancer [112,113,114]. Tang et al. demonstrated that Ca^2+^ release via MCU is critical for SOCE-promoted metastasis in MDA-MB-231 breast cancer cells [112]. By contrast, Tosatto et al. suggested that the distinctive role of MCU enhances breast migration progression via a mechanism involving hypoxia-inducible factor-1α (HIF-1α) signalling, and they attributed the indirect effects of MCU on Ca^2+^ signalling via SOCE that was observed by Tan et al. to the cell line-dependent effect [113]. Similarly, recent evidence by Wang et al. is consistent with Tosatto et al.’s finding that MCU-mediated mitochondrial Ca^2+^ signals enhance metastatic phenotypes (involving the epithelial-mesenchymal transition process) through a distinctive mechanism via HIF-1α and VEGF (Vascular endothelial growth factor) signalling pathways in gastric cancer [115]. What remains unanswered is how MCU acts at the molecular level and what the possible complex interplay is between mitochondrial Ca^2+^ signalling, SOCE and metastatic cancer. These factors warrant further investigation in various cancer subtypes utilising in vitro and in vivo models.

### 3.3. Voltage-Gated Ca^2+^ Channels in Metastasis

Recently, voltage-gated Ca^2+^ channels (VGCCs), particularly L and T subtypes, have been implicated in the pathophysiological processes that drive cancer metastasis [116,117,118,119,120,121]. Grasset et al. demonstrated that pharmacological inhibition of the L-type calcium channel via verapamil or diltiazem decreases the EGF signalling mediated collective cancer cell invasion in in vitro and in vivo models of squamous cell carcinoma [120]. Recent evidence provided by Phiwchai et al. (2020) revealed the involvement of L-type calcium channel/Ca^2+^ signalling pathway in labile iron-driving hepatic cancer cell proliferation [121]. Knocked down or pharmacologically inhibited T-type calcium channels showed reduced migration and invasion of BRAFV600E cells, which provides evidence that T-type calcium channels play a role in melanoma metastasis [118]. These data highlight the potential of these channels to serve as promising therapeutic targets to treat patients with metastatic carcinomas due to the long-term medical use of these channel.

## 4. Proteins Involved in Ca^2+^ Signalling Cascades and Their Roles in Metastasis

The crosstalk between calcium effector proteins such as calpain and calmodulin (CaM), and endolysosomal proteins such as the lysosome-associated membrane proteins (LAMPs), and cancer metastasis has begun to be unravelled. There are 15 isoforms of the calpain family of calcium-dependent cysteine proteases in mammals [122] and of those isoforms, calpain-1, calpain-2 and calpain-9 have received considerable scientific attention for their roles in metastatic traits [123]. An increased expression of calpain-1 was detected in colorectal cancer and correlated with poor overall survival (OS), advanced pathological grade, and metastasis [124]. Calpain-1 deficient SW480 and HT29 cells (an in vitro model of colorectal cancer achieved by siRNA) exhibited significantly reduced of cell invasion and migration processes, which ultimately promoted tumour progression and metastasis compared to controlled cells [124]. Similarly, Yu et al. found that upregulation of calpain-1 protein levels were significantly associated with tumour progression and shorter OS in patients with pancreatic cancer [125]. When calpain-1 expression in pancreatic cancer cells was downregulated by siRNA in AsPC-1 and BxPC-3 cell lines, the invasion and migration abilities of pancreatic cancer cells were significantly attenuated [38]. Previously, calpain-1 overexpression was significantly associated with gallbladder carcinoma compared to cholecystitis, indicating that calpain-1 might act as a key mediator shifting gallbladder cells towards a tumour progression state that would make it a clinical tool for gallbladder carcinoma prognosis [126].

In 2003, Mamone, et al. discovered the emerging roles of calpain-2 at epigenetic levels, using in vitro and in vivo prostate cancer models as potential therapeutic targets to hinder metastatic prostate cancer [127]. These findings are consistent with a recent study conducted by Gao et al. that identified elevated levels of calpain-2 proteins in metastatic prostate cancer compared to primary tumours [128]. They also deciphered the underlying molecular mechanism of epigenetic activation for calpain-2-evoked cancer metastasis via the nuclear factor- κB (NF-κB)/ DNA (cytosine-5)-methyltransferase 1(DNMT1) signalling pathway [128].

In contrast to calpain-1 and calpain-2 isoforms, the downregulation of calpain-9 expression was associated with metastasis in patients with gastric cancer, suggesting the protective effect of calpain-9 expression and its roles in hampering gastric cancer progression [129]. Calpain small subunit 1 (Capn4) acts as a maintainer of calpain function and belongs to the calpain family. A growing body of evidence has demonstrated its promising prognostic biomarker potential and the crucial roles of Capn4 in metastatic phenotypes, from tumour invasion to progression, in various types of cancer that include nasopharyngeal carcinoma, gastric cancer, ovarian carcinoma, breast cancer, glioma and oesophageal squamous cell carcinoma [130,131,132,133,134,135]. Capn4 exhibited distinct underlying mechanisms depending on the cancer subtype context. The precise mechanisms underlying the actions of Capn4 and its complex interplay between Epstein-Barr virus latent membrane protein 1 (LMP1) and nasopharyngeal carcinoma metastasis, was uncovered via enhanced actin rearrangement-mediated ERK/JNK/AP-1 pathway signalling [130]. In addition, Zhao, et al. found that Capn4 promoted-cell invasion and gastric cancer metastasis involving Wnt/β-catenin/MMP9 signalling [134].

Calmodulin (CaM) is a multifunctional Ca^2+^ binding protein. Its role in metastatic traits was recently reviewed by Villalobo, and Martin, providing valuable insight into the roles of calmodulin in metastasis, from invasiveness to tumour cell migration [136]. It was shown that calcium/calmodulin-dependent protein kinase II (CaMKII) triggered gastric cancer cell metastasis by activating nuclear factor-κB (NF-κB) signalling involving AKT, which ultimately enhanced MMP-9production in BGC-803 cells (an in vitro model of human gastric cancer) [137]; this is a metastatic prompting protein present in various cancer subtypes. Pharmacological modulation of CaM by KN93, a specific inhibitor, in HCT116 cells (an in vitro model of human colon cancer) was found to drastically decrease colon cancer cell invasion and migration via ERK1/2 or p38 signalling [138]. Acetyl-CoA- activates cytosolic CaMKII-mediated metastasis in in vitro and in vivo models of prostate cancer [139].

The lysosome-associated membrane protein (LAMP) family consists of five members expressed mainly in the lysosome [140]. LAMP proteins are involved in various aspects of cancer metastasis biology. They maintain lysosomal homeostasis, where much endolysosomal Ca^+2^ signalling occurs. Although it has become clear that lysosome-associated membrane proteins play significant roles in autophagy [141], which contributes to cancer metastasis [142], the complex interplay between LAMPs, Ca^2+^ signals, and autophagy-mediated metastasis remains elusive. LAMP1, LAMP2, and LAMP3 are the key LAMP isoforms emerging as important potential players in cancer biology [140]. Upregulation of LAMP1 expression has been reported to predict poor prognosis in various cancer subtypes including large B-cell lymphoma, epithelial ovarian cancer, breast cancer, and laryngeal squamous cell carcinoma [143,144,145,146]. The underlying mechanism of the role that ubiquitin-like protein 4A (UBL4A) plays in autophagy-mediated metastasis by suppressing autophagy and disturbing lysosomal functions through targeting LAMP1 in pancreatic ductal adenocarcinoma was unveiled recently [147]. Overexpression of LAMP2 has been associated with worse OS in oesophageal squamous cell carcinoma patients [148]. Upregulation of LAMP3 expression acts as a biomarker for poor prognosis in oesophageal squamous cell carcinoma (ESCC) and ovarian cancer [149,150], whereas downregulation of LAMP3 expression has been associated with poor prognosis in hepatocellular carcinoma [151]. A recent study conducted by Huang et al. provides a possible explanation for LAMP3 overexpression contributing to poor prognosis in ESCC [152]. The authors found that LAMP3-deficient ESCC cells had drastically reduced metastatic traits (invasive and metastatic capability) compared to non-deficient ESCC cells via activation of the cAMP-dependent protein kinase A (PKA)-mediated VASP phosphorylation pathway [152]. In addition, the authors showed that the number of lung metastases were attenuated after LAMP3 knockdown in an in vivo mouse model used for investigating LAMP3-mediated ESCC cell metastasis [152]. These findings imply that the proteins involved in Ca^2+^ signalling or lysosomal function fulfil functions far beyond their roles in maintaining Ca^2+^ or lysosomal homeostasis. Study of the interaction of these proteins in the context of metastasis might form the basis of a fruitful therapeutic approach for metastatic cancer. Further work is required to uncover the communication between LAMPs and Ca^2+^ signalling in lysosomes at a dynamic level.

## 5. Challenges and Potential Clinical Utilities of Calcium Signalling as a Diagnostic and Therapeutic Target in Metastatic Cancer

Despite significant advances in the current approaches to diagnosing and treating metastatic cancer in clinical settings, some patients still have low successful response rates to therapy or experience delay in the detection of metastatic sites; hence identifying innovative biomarkers and therapeutic targets for metastatic cancer detection or therapy is required. Molecular characterization of Ca^2+^ signalling’s role in cell invasion and motility, tumour progression, and metastasis is an evolving field receiving increased scientific attention, raising important questions regarding the possibility of translating these findings into potential clinical tools to optimize metastatic cancer diagnosis and therapy. While navigating clinicaltrials.gov, we found a paucity of clinical studies using changes in Ca^2+^ signalling pathways as a detection approach for metastatic cancer or targeting Ca^2+^ proteins as an adjuvant therapeutic approach for patients with metastatic cancer. Calcium electroporation (CaEP), characterized by introducing supraphysiological calcium concentrations into cells by applying electrical pulses [153], is a promising novel adjuvant therapeutic approach for cancer patients. This strategy is currently under investigation in phase 2 clinical trials (such as NCT01941901, NCT04259658, and NCT03628417), mainly in skin cancers in the metastatic state, in which it is administered intratumourally. A phase 1 clinical trial (NCT01056029) was conducted to investigate mipsagargin, which is a thapsigargin (noncompetitive inhibitor of the sarco-/endoplasmic reticulum Ca^2+^ ATPase) pro-drug, in locally advanced or metastatic solid tumours. Generally, mipsagargin has been shown to have acceptable safety and tolerability profiles, with prolonged disease stabilisation in some patients with solid tumours [154]. Mipsagargin has moved into phase 2, and its investigation has been completed in various cancer subtypes including hepatocellular carcinoma (NCT01777594), glioblastoma (NCT02067156), clear cell renal cell carcinoma (NCT02607553), and prostatic neoplasms (NCT02381236). In a phase 1 clinical trial (NCT01480050), combination therapy of mibefradil dihydrochloride (a T-type calcium channel blocker) and temozolomide (an alkylating agent) in patients with recurrent advanced stages of gliomas was found to be well-tolerated, with encouraging clinical responses in a subset of patients [155], warranting further investigation in phase 2 trials. Despite Ca^2+^ being a ubiquitous second messenger, defining distinct downstream/upstream regulators of Ca^2+^ signalling pathways could be used to provide potential translation of preclinical evidence into clinical studies, in order to ultimately develop more effective and less toxic chemotherapeutic agents.

## 6. Conclusions

In summary, a growing body of evidence reveals the substantial effects of Ca^2+^ signalling-mediated cancer metastasis, raising important questions regarding the clinical utility of proteins involved in Ca^2+^ signalling cascades as cancer biomarkers or hallmarks. Several studies have detected dysregulated expression of intracellular or extracellular calcium channels or proteins related to Ca^2+^ signalling-triggered metastasis at the mRNA or protein levels in various cancer subtypes (see Table 1). These are attributed to pathophysiological processes, including cellular adhesion, motility, invasion, the epithelial-mesenchymal transition, and cell progression and migration at a local level, as well as the development of metastasis at a systemic level. Accumulating evidence points to an association between calcium channel proteins or Ca^2+^ signalling-related proteins at the mRNA or protein levels and the prognosis of patients with different types of cancers, suggesting possible clinical applications of Ca^2+^ signalling proteins as prognostic biomarkers. However, large prospective clinical studies with diverse patient populations are required to validate these findings and sufficiently establish the specificity and sensitivity of these biomarkers for cancer at a global level or among different cancers for them to be employed in our daily clinical practice.

To date, a few clinical trials have investigated the pharmacological modulation of Ca^2+^ signalling as a therapeutic strategy to treat patients with metastatic cancer. Calcium electroporation, mipsagargin and mibefradil in combination with temozolomide showed promising results in the early stages of clinical trials, warranting further investigation. This supports the possibility of translating these therapeutic strategies into the clinic as novel alternative approaches to be given alone or as adjuvants with other chemotherapeutic agents if they pass the development stages and are approved for clinical use by federal agencies, such as the Food and Drug Administration (FDA) or the European Medicines Agency (EMA). Despite the emerging roles of Ca^2+^ signalling in tumour progression and metastasis and its potential as a clinical tool that can enhance the detection rate and guide the treatment of metastatic cancer patients, several questions still remain to be answered, such as those relating to the precise mechanisms underlying Ca^2+^ signalling-mediated cancer metastasis. A key diagnostic or therapeutic challenge is discovering specific downstream or upstream regulators of Ca^2+^ signalling that are involved in metastatic cascades given the ubiquity of Ca^2+^ signals in our cells. Ca^2+^ signalling pathways are involved in diverse aspects of tumour progression and metastasis, and this further research will open up the possibility of using Ca^2+^ proteins as clinical biomarkers and utilising pharmacological modulators to optimise metastatic cancer therapy.

## Figures and Tables

**Table 1:** Some experimental evidence supporting Ca^+2^ signalling-mediated cancer metastasis.

Target				Expression		Type of Cancer	Process Related to Metastasis	Mechanism (If Applicable)	In Vitro (Cell Line)/In Vivo	Ref.
IP3R3	Intracellular calcium signalling in metastasis	Endoplasmic and sarcoplasmic reticulum Ca^2+^ channels/pumps	IP_3_ receptors (IP_3_Rs)	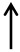	Increased mRNA and protein levels	Breast cancer	Migration	Ca^2+^ signalling via IP_3_R3 mediated cancer cell metastasis	MDA-MB-231 and MDA-MB-435S cells	[24]
				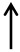	Increased protein levels	Cholangiocarcinoma (CCA)	Migration		Patients with hilar/intrahepatic CCA and CCA cell lines	[25]
				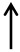	Increased protein levels	Colorectal carcinoma	Aggressiveness		Patients with advanced/metasatic colorectal carcinoma	[27]
				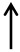	Increased mRNA levels	Glioblastoma	Invasion and migration		Patients with glioblastoma	[28]
RYR2			Ryanodine receptors (RyRs)	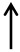	Increased mRNA levels	Breast cancer	Epithelial-mesenchymal transition (EMT)	RYR2/Ca^2+^ signals activate EGF-mediated EMT	MDA-MB-468 cells	[20]
TRAM2			Translocons	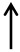	Increased mRNA levels	Oral squamous cell carcinoma (OSCC)	Cellular invasion, and migration	Overexpression of TRAM2-mediated matrix metalloproteinase activation	OSCC-derived cell lines and primary OSCC tissues	[21]
SERCA2			Sarco-endoplasmic reticulum Ca^2+^ reuptake pump (SERCA)	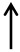	Increased protein levels	Colorectal Cancer (CRC)	Progression	Calcium signalling via SERCA2 mediation CRC progression	Patients with advanced stages of colorectal cancer	[16]
TPCs		Endolysosomal Ca^2+^ Channels	Two-pore channels (TPCs)	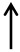	Increased TPC1/TPC2 mRNA levels	Bladder cancer	Cell adhesion and migration	Endolysosomal Ca^2+^ signaling via TPC evoked β1-integrin recycling	T24 cells	[31]
TPC2				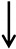	Decreased TPC2 mRNA levels	Melanoma	Cell adhesion and invasion	Reduction in TPC2 expression enhanced metastasis via YAP/TAZ activation	Patients with metastatic skin cutaneous melanoma (SKCM)	[32]
TRPML1			Transient receptor potential cation channels (TRPMLs)	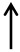	Increased mRNA levels	Non- small-cell lung cancer (NSCLC)	Invasion and migration	Ca^2+^ signals via TRPML1- mediated autophagy promoting tumor progression	Patients with advanced-stage ( III–IV) NSCLC	[35]
TRPML2				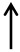	Increased mRNA and protein levels	Glioma	Cell proliferation and progression	Ca^2+^ signalling via TRPML2 promoting Glioma progression	Patients with advanced-stage (III–IV) glioma	[36]
CaSR	Extracellular components of Ca^2+^ signaling in metastasis	Calcium-sensing receptor (CaSR)		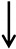	Decreased mRNA level	Colorectal cancer (CRC)	Cell proliferation, differentiation and apoptosis	/	HT29/Caco2-15/colorectal cancer patients	[58]
				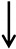	Decreased mRNA and protein level	Parathyroid cancer	Cell proliferation	CaSR activation increases ERK phosphorylation	Patients with parathyroid adenomas	[59]
				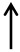	Increased mRNA and protein level	Breast cancer	Cell proliferation and migration	ERK1/2 MAPK or phospholipase Cβ (PLCβ) pathway	Patients with breast cancer/breast cancer cell lines MDA-MB-231, MCF7, T47D, and BT474	[62,63]
				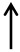	Increased protein level	Prostate cancer	Cell proliferation and migration	CaSR mediated cell attachmentvia the AKT signaling pathway	Human prostate cancer tissue sections/prostate celllines PC-3, C4-2B and LNCaP	[57,61]
				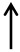	Increased mRNA and protein level	renal cell carcinoma (RCC)	Cell proliferation and migration	CaSR activated the PI3K (phospatidyl-inositol 3-kinase)/AKT, PLCγ-1, and MAPK pathway	Primary cells derived from RCC patients	[65]
ORAI1		Store-operated calcium entry (SOCE)	ORAI	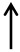	Increased protein level	Melanoma	Cell proliferation and migration	SOCE increases phosphorylation of ERK and calpain-dependent actin dynamics	Human metastatic melanoma cell lines	[68]
				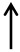	Increased mRNA and protein levels	Hepatocarcinoma (HCC)	Autophagic cell death	Orai1 blocks autophagy through AKT/mTOR signalling pathway	Tissues from HCC patients and human hepatocarcinoma cell line HepG2	[73]
ORAI 1 & ORAI 2				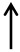	Increased mRNA and protein levels	Acute myeloid leukemia	Cell proliferation and migration	Promoting phosphorylation of the focal adhesion kinase (FAK)	HL60 cell line	[66]
ORAI 3				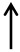	Increased mRNA and protein levels	Breast cancer	Cell proliferation and migration	SOCE-dependent NFAT activity and ERK1/2 and FAK kinase phosphorylation	MCF7 and MDA-MB231 cell lines/in vivo	[75]
STIM1			Stromal-interaction molecule (STIM)	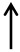	Increased protein level	Cervical cancer	Cell growth, migration, and angiogenesis	STIM1 activate calpain and Pyk2, which regulate FAK	Human cervical cancer cell lines SiHa and CaSki/in vivo	[76]
STIM1				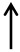	Increased mRNA and protein levels	Gastric cancer	Cell migration and invasion	/	Human gastric cancer cells/gastric tumor tissues	[77]
TRPM8			TRP channels	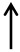	Increased mRNA	Bladder cancer	Cell proliferation and migration	Ca^2+^ signalling via TRPM8 mediated bladder cancer cell metastasis	Human bladder cancer tissue	[101]
TRPV4				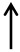	Increased protein level	Gastric cancer	Cell proliferation and invasion	TRPV4/Ca^2+^ signalling-mediated EMT	Human gastric cancer tissues	[108]

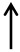
 Increased; 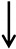 Decreased.

## References

[1] Chaffer C.L., Weinberg R.A. (2011). A perspective on cancer cell metastasis. Science.

[2] Tsai F.C., Kuo G.H., Chang S.W., Tsai P.J. (2015). Ca^2+^ signaling in cytoskeletal reorganization, cell migration, and cancer metastasis. BioMed Res. Int..

[3] Nielsen N., Lindemann O., Schwab A. (2014). TRP channels and STIM/ORAI proteins: Sensors and effectors of cancer and stroma cell migration. Br. J. Pharmacol..

[4] Chen Y.F., Chen Y.T., Chiu W.T., Shen M.R. (2013). Remodeling of calcium signaling in tumor progression. J. Biomed. Sci..

[5] Iamshanova O., Fiorio Pla A., Prevarskaya N. (2017). Molecular mechanisms of tumour invasion: Regulation by calcium signals. J. Physiol..

[6] White C. (2017). The regulation of tumor cell invasion and metastasis by endoplasmic reticulum-to-mitochondrial Ca^2+^ transfer. Front. Oncol..

[7] Parkash J., Asotra K. (2010). Calcium wave signaling in cancer cells. Life Sci..

[8] Chen Y.F., Hsu K.F., Shen M.R. (2016). The store-operated Ca^2+^ entry-mediated signaling is important for cancer spread. Biochim. Biophys. Acta (BBA) Mol. Cell Res..

[9] Alharbi A.F., Parrington J. (2019). Endolysosomal Ca^2+^ Signaling in Cancer: The Role of TPC2, from Tumorigenesis to Metastasis. Front. Cell Dev. Biol..

[10] Tajbakhsh A., Pasdar A., Rezaee M., Fazeli M., Soleimanpour S., Hassanian S.M., FarshchiyanYazdi Z., Younesi Rad T., Ferns G.A., Avan A. (2018). The current status and perspectives regarding the clinical implication of intracellular calcium in breast cancer. J. Cell. Physiol..

[11] Grimm C., Bartel K., Vollmar A.M., Biel M. (2018). Endolysosomal cation channels and cancer—A link with great potential. Pharmaceuticals.

[12] Faris P., Shekha M., Montagna D., Guerra G., Moccia F. (2019). Endolysosomal Ca^2+^ signalling and cancer hallmarks: Two-pore channels on the move, TRPML1 lags behind!. Cancers.

[13] Tharmalingam S., Hampson D.R. (2016). The calcium-sensing receptor and integrins in cellular differentiation and migration. Front. Physiol..

[14] Mo P., Yang S. (2018). The store-operated calcium channels in cancer metastasis: From cell migration, invasion to metastatic colonization. Front. Biosci. (Landmark Ed.).

[15] Primeau J.O., Armanious G.P., M’Lynn E.F., Young H.S. (2018). The sarcoendoplasmic reticulum calcium ATPase. Membrane Protein Complexes: Structure and Function.

[16] Chung F.Y., Lin S.R., Lu C.Y., Yeh C.S., Chen F.M., Hsieh J.S., Huang T.J., Wang J.Y. (2006). Sarco/endoplasmic reticulum calcium-ATPase 2 expression as a tumor marker in colorectal cancer. Am. J. Surg. Pathol..

[17] Xu X.Y., Gou W.F., Yang X., Wang G.L., Takahashi H., Yu M., Mao X.Y., Takano Y., Zheng H.C. (2012). Aberrant SERCA3 expression is closely linked to pathogenesis, invasion, metastasis, and prognosis of gastric carcinomas. Tumor Biol..

[18] Shi C., Cai Y., Li Y., Li Y., Hu N., Ma S., Hu S., Zhu P., Wang W., Zhou H. (2018). Yap promotes hepatocellular carcinoma metastasis and mobilization via governing cofilin/F-actin/lamellipodium axis by regulation of JNK/Bnip3/SERCA/CaMKII pathways. Redox Biol..

[19] Rondé P., Giannone G., Gerasymova I., Stoeckel H., Takeda K., Haiech J. (2000). Mechanism of calcium oscillations in migrating human astrocytoma cells. Biochim. Biophys. Acta (BBA) Mol. Cell Res..

[20] Davis F.M., Parsonage M.T., Cabot P.J., Parat M.O., Thompson E.W., Roberts-Thomson S.J., Monteith G.R. (2013). Assessment of gene expression of intracellular calcium channels, pumps and exchangers with epidermal growth factor-induced epithelial-mesenchymal transition in a breast cancer cell line. Cancer Cell Int..

[21] Fukushima R., Kasamatsu A., Nakashima D., Higo M., Fushimi K., Kasama H., Endo-Sakamoto Y., Shiiba M., Tanzawa H., Uzawa K. (2018). Overexpression of translocation associated membrane protein 2 leading to cancer-associated matrix metalloproteinase activation as a putative metastatic factor for human oral cancer. J. Cancer.

[22] Paknejad N., Hite R.K. (2018). Structural basis for the regulation of inositol trisphosphate receptors by Ca^2+^ and IP 3. Nat. Struct. Mol. Biol..

[23] Hedberg M.L., Goh G., Chiosea S.I., Bauman J.E., Freilino M.L., Zeng Y., Wang L., Diergaarde B.B., Gooding W.E., Lui V.W. (2016). Genetic landscape of metastatic and recurrent head and neck squamous cell carcinoma. J. Clin. Investig..

[24] Mound A., Vautrin-Glabik A., Foulon A., Botia B., Hague F., Parys J.B., Ouadid-Ahidouch H., Rodat-Despoix L. (2017). Downregulation of type 3 inositol (1, 4, 5)-trisphosphate receptor decreases breast cancer cell migration through an oscillatory Ca^2+^ signal. Oncotarget.

[25] Ueasilamongkol P., Khamphaya T., Guerra M.T., Rodrigues M.A., Gomes D.A., Kong Y., Wei W., Jain D., Trampert D.C., Ananthanarayanan M. (2020). Type 3 inositol 1, 4, 5-trisphosphate receptor is increased and enhances malignant properties in cholangiocarcinoma. Hepatology.

[26] Sakakura C., Hagiwara A., Fukuda K., Shimomura K., Takagi T., Kin S.H., Nakase Y., Fujiyama J., Mikoshiba K., Okazaki Y. (2003). Possible involvement of inositol 1,4,5-trisphosphate receptor type 3 (IP3R3) in the peritoneal dissemination of gastric cancers. Anticancer Res..

[27] Shibao K., Fiedler M.J., Nagata J., Minagawa N., Hirata K., Nakayama Y., Iwakiri Y., Nathanson M.H., Yamaguchi K. (2010). The type III inositol 1,4,5-trisphosphate receptor is associated with aggressiveness of colorectal carcinoma. Cell Calcium.

[28] Kang S.S., Han K.S., Ku B.M., Lee Y.K., Hong J., Shin H.Y., Almonte A.G., Woo D.H., Brat D.J., Hwang E.M. (2010). Caffeine-mediated inhibition of calcium release channel inositol 1, 4, 5-trisphosphate receptor subtype 3 blocks glioblastoma invasion and extends survival. Cancer Res..

[29] Iommelli F., De Rosa V., Terlizzi C., Monti M., Panico M., Fonti R., Del Vecchio S. (2018). Inositol Trisphosphate Receptor Type 3-mediated Enhancement of EGFR and MET Cotargeting Efficacy in Non–Small Cell Lung Cancer Detected by 18F-fluorothymidine. Clin. Cancer Res..

[30] Huang X., Jin M., Chen Y.X., Wang J., Zhai K., Chang Y., Yuan Q., Yao K.T., Ji G. (2016). ERP44 inhibits human lung cancer cell migration mainly via IP3R2. Aging (Albany N. Y.).

[31] Nguyen O.N., Grimm C., Schneider L.S., Chao Y.K., Atzberger C., Bartel K., Watermann A., Ulrich M., Mayr D., Wahl-Schott C. (2017). Two-pore channel function is crucial for the migration of invasive cancer cells. Cancer Res..

[32] D’Amore A., Hanbashi A.A., Di Agostino S., Palombi F., Sacconi A., Voruganti A., Taggi M., Canipari R., Blandino G., Parrington J. (2020). Loss of Two-Pore Channel 2 (TPC2) Expression Increases the Metastatic Traits of Melanoma Cells by a Mechanism Involving the Hippo Signalling Pathway and Store-Operated Calcium Entry. Cancers.

[33] Jaślan D., Böck J., Krogsaeter E., Grimm C. (2020). Evolutionary Aspects of TRPMLs and TPCs. Int. J. Mol. Sci..

[34] Lyu L., Jin X., Li Z., Liu S., Li Y., Su R., Su H. (2020). TBBPA regulates calcium-mediated lysosomal exocytosis and thereby promotes invasion and migration in hepatocellular carcinoma. Ecotoxicol. Environ. Saf..

[35] Yin C., Zhang H., Liu X., Zhang H., Zhang Y., Bai X., Wang L., Li H., Li X., Zhang S. (2019). Downregulated MCOLN1 attenuates the progression of non-small-cell lung cancer by inhibiting lysosome-autophagy. Cancer Manag. Res..

[36] Morelli M.B., Nabissi M., Amantini C., Tomassoni D., Rossi F., Cardinali C., Santoni M., Arcella A., Oliva M.A., Santoni A. (2016). Overexpression of transient receptor potential mucolipin-2 ion channels in gliomas: Role in tumor growth and progression. Oncotarget.

[37] Wu M., Li X., Zhang T., Liu Z., Zhao Y. (2019). Identification of a nine-gene signature and establishment of a prognostic nomogram predicting overall survival of pancreatic cancer. Front. Oncol..

[38] Murrell-Lagnado R.D. (2018). A role for P2X4 receptors in lysosome function. J. Gen. Physiol..

[39] Garrity A.G., Wang W., Collier C.M., Levey S.A., Gao Q., Xu H. (2016). The endoplasmic reticulum, not the pH gradient, drives calcium refilling of lysosomes. Elife.

[40] Haren N., Khorsi H., Faouzi M., Ahidouch A., Sevestre H., Ouadid-Ahidouch H. (2010). Intermediate conductance Ca^2+^ activated K+ channels are expressed and functional in breast adenocarcinomas: Correlation with tumour grade and metastasis status. Histol. Histopathol..

[41] Tajima N., Schönherr K., Niedling S., Kaatz M., Kanno H., Schönherr R., Heinemann S.H. (2006). Ca^2+^-activated K+ channels in human melanoma cells are up-regulated by hypoxia involving hypoxia-inducible factor-1α and the von Hippel-Lindau protein. J. Physiol..

[42] Rabjerg M., Oliván-Viguera A., Hansen L.K., Jensen L., Sevelsted-Møller L., Walter S., Jensen B.L., Marcussen N., Köhler R. (2015). High expression of KCa3. 1 in patients with clear cell renal carcinoma predicts high metastatic risk and poor survival. PLoS ONE.

[43] Thurber A.E., Nelson M., Frost C.L., Levin M., Brackenbury W.J., Kaplan D.L. (2017). IK channel activation increases tumor growth and induces differential behavioral responses in two breast epithelial cell lines. Oncotarget.

[44] Vergara C., Latorre R., Marrion N.V., Adelman J.P. (1998). Calcium-activated potassium channels. Curr. Opin. Neurobiol..

[45] Catacuzzeno L., Aiello F., Fioretti B., Sforna L., Castigli E., Ruggieri P., Tata A.M., Calogero A., Franciolini F. (2011). Serum-activated K and Cl currents underlay U87-MG glioblastoma cell migration. J. Cell. Physiol..

[46] d’Alessandro G., Catalano M., Sciaccaluga M., Chece G., Cipriani R., Rosito M., Grimaldi A., Lauro C., Cantore G., Santoro A. (2013). KCa3. 1 channels are involved in the infiltrative behavior of glioblastoma in vivo. Cell Death Dis..

[47] Rosa P., Catacuzzeno L., Sforna L., Mangino G., Carlomagno S., Mincione G., Petrozza V., Ragona G., Franciolini F., Calogero A. (2018). BK channels blockage inhibits hypoxia-induced migration and chemoresistance to cisplatin in human glioblastoma cells. J. Cell. Physiol..

[48] Rosa P., Sforna L., Carlomagno S., Mangino G., Miscusi M., Pessia M., Franciolini F., Calogero A., Catacuzzeno L. (2017). Overexpression of large-conductance calcium-activated potassium channels in human glioblastoma stem-like cells and their role in cell migration. J. Cell. Physiol..

[49] Ruggieri P., Mangino G., Fioretti B., Catacuzzeno L., Puca R., Ponti D., Miscusi M., Franciolini F., Ragona G., Calogero A. (2012). The inhibition of KCa3. 1 channel activity reduces cell motility in glioblastoma-derived cancer stem cells. PLoS ONE.

[50] Klumpp L., Sezgin E.C., Skardelly M., Eckert F., Huber S.M. (2018). KCa3. 1 channels and glioblastoma: In vitro studies. Curr. Neuropharmacol..

[51] D’Alessandro G., Monaco L., Catacuzzeno L., Antonangeli F., Santoro A., Esposito V., Franciolini F., Wulff H., Limatola C. (2019). Radiation increases functional KCa3. 1 expression and invasiveness in glioblastoma. Cancers.

[52] Edalat L., Stegen B., Klumpp L., Haehl E., Schilbach K., Lukowski R., Kühnle M., Bernhardt G., Buschauer A., Zips D. (2016). BK K+ channel blockade inhibits radiation-induced migration/brain infiltration of glioblastoma cells. Oncotarget.

[53] Hynes R.O. (2009). The extracellular matrix: Not just pretty fibrils. Science.

[54] Gopal S., Multhaupt H.A.B., Couchman J.R. (2020). Calcium in Cell-Extracellular Matrix Interactions. Adv. Exp. Med. Biol..

[55] D’Souza-Li L. (2006). The calcium-sensing receptor and related diseases. Arq. Bras. Endocrinol. Metabol..

[56] Vezzoli G., Soldati L., Gambaro G. (2009). Roles of calcium-sensing receptor (CaSR) in renal mineral ion transport. Curr. Pharm. Biotechnol..

[57] Liao J., Schneider A., Datta N.S., McCauley L.K. (2006). Extracellular calcium as a candidate mediator of prostate cancer skeletal metastasis. Cancer Res..

[58] Aggarwal A., Prinz-Wohlgenannt M., Tennakoon S., Höbaus J., Boudot C., Mentaverri R., Brown E.M., Baumgartner-Parzer S., Kállay E. (2015). The calcium-sensing receptor: A promising target for prevention of colorectal cancer. Biochim. Biophys. Acta (BBA) Mol. Cell Res..

[59] Corbetta S., Mantovani G., Lania A., Borgato S., Vicentini L., Beretta, Faglia G., Di Blasio A.M., Spada A. (2000). Calcium-sensing receptor expression and signalling in human parathyroid adenomas and primary hyperplasia: Calcium-sensing receptor in parathyroid tumours. Clin. Endocrinol..

[60] Tennakoon S., Aggarwal A., Kállay E. (2016). The calcium-sensing receptor and the hallmarks of cancer. Biochim. Biophys. Acta (BBA) Mol. Cell Res..

[61] Feng J., Xu X., Li B., Brown E., Farris A.B., Sun S.-Y., Yang J.J. (2014). Prostate cancer metastatic to bone has higher expression of the calcium-sensing receptor (CaSR) than primary prostate cancer. Recept. Clin. Investig..

[62] Mihai R., Stevens J., McKinney C., Ibrahim N.B.N. (2006). Expression of the calcium receptor in human breast cancer—A potential new marker predicting the risk of bone metastases. Eur. J. Surg. Oncol..

[63] Saidak Z., Boudot C., Abdoune R., Petit L., Brazier M., Mentaverri R., Kamel S. (2009). Extracellular calcium promotes the migration of breast cancer cells through the activation of the calcium sensing receptor. Exp. Cell Res..

[64] Li H.-X., Kong F.-J., Bai S.-Z., He W., Xing W.-J., Xi Y.-H., Li G.-W., Guo J., Li H.-Z., Wu L.-Y. (2012). Involvement of calcium-sensing receptor in oxLDL-induced MMP-2 production in vascular smooth muscle cells via PI3K/Akt pathway. Mol. Cell. Biochem..

[65] Joeckel E., Haber T., Prawitt D., Junker K., Hampel C., Thüroff J.W., Roos F.C., Brenner W. (2014). High calcium concentration in bones promotes bone metastasis in renal cell carcinomas expressing calcium-sensing receptor. Mol. Cancer.

[66] Diez-Bello R., Jardin I., Salido G.M., Rosado J.A. (2017). Orai1 and Orai2 mediate store-operated calcium entry that regulates HL60 cell migration and FAK phosphorylation. Biochim. Biophys. Acta (BBA) Mol. Cell Res..

[67] Putney J.W. (2009). Capacitative calcium entry: From concept to molecules. Immunol. Rev..

[68] Umemura M., Baljinnyam E., Feske S., De Lorenzo M.S., Xie L.-H., Feng X., Oda K., Makino A., Fujita T., Yokoyama U. (2014). Store-Operated Ca^2+^ Entry (SOCE) Regulates Melanoma Proliferation and Cell Migration. PLoS ONE.

[69] Stanisz H., Saul S., Müller C.S.L., Kappl R., Niemeyer B.A., Vogt T., Hoth M., Roesch A., Bogeski I. (2014). Inverse regulation of melanoma growth and migration by Orai1/STIM2-dependent calcium entry. Pigment Cell Melanoma Res..

[70] El Boustany C., Bidaux G., Enfissi A., Delcourt P., Prevarskaya N., Capiod T. (2008). Capacitative calcium entry and transient receptor potential canonical 6 expression control human hepatoma cell proliferation. Hepatology.

[71] Feske S. (2010). CRAC channelopathies. Pflug. Arch. Eur. J. Physiol..

[72] Sullivan R.J., Atkins M.B. (2010). Molecular targeted therapy for patients with melanoma: The promise of MAPK pathway inhibition and beyond. Expert Opin. Investig. Drugs.

[73] Tang B.-D., Xia X., Lv X.-F., Yu B.-X., Yuan J.-N., Mai X.-Y., Shang J.-Y., Zhou J.-G., Liang S.-J., Pang R.-P. (2017). Inhibition of Orai1-mediated Ca^2+^ entry enhances chemosensitivity of HepG2 hepatocarcinoma cells to 5-fluorouracil. J. Cell. Mol. Med..

[74] Chakraborty S., Ghosh S., Banerjee B., Santra A., Adhikary A., Misra A.K., Sen P.C. (2016). Phemindole, a Synthetic Di-indole Derivative Maneuvers the Store Operated Calcium Entry (SOCE) to Induce Potent Anti-Carcinogenic Activity in Human Triple Negative Breast Cancer Cells. Front. Pharmacol..

[75] Motiani R.K., Zhang X., Harmon K.E., Keller R.S., Matrougui K., Bennett J.A., Trebak M. (2013). Orai3 is an estrogen receptor α-regulated Ca^2+^ channel that promotes tumorigenesis. FASEB J..

[76] Chen Y.-F., Chiu W.-T., Chen Y.-T., Lin P.-Y., Huang H.-J., Chou C.-Y., Chang H.-C., Tang M.-J., Shen M.-R. (2011). Calcium store sensor stromal-interaction molecule 1-dependent signaling plays an important role in cervical cancer growth, migration, and angiogenesis. Proc. Natl. Acad. Sci. USA.

[77] Xu J.-M., Zhou Y., Gao L., Zhou S.-X., Liu W.-H., Li X.-A. (2016). Stromal interaction molecule 1 plays an important role in gastric cancer progression. Oncol. Rep..

[78] Moran M.M. (2018). TRP channels as potential drug targets. Annu. Rev. Pharmacol. Toxicol..

[79] Cuddapah V.A., Turner K.L., Sontheimer H. (2013). Calcium entry via TRPC1 channels activates chloride currents in human glioma cells. Cell Calcium.

[80] Guéguinou M., Harnois T., Crottes D., Uguen A., Deliot N., Gambade A., Chantôme A., Haelters J.P., Jaffrès P.A., Jourdan M.L. (2016). SK3/TRPC1/Orai1 complex regulates SOCE-dependent colon cancer cell migration: A novel opportunity to modulate anti-EGFR mAb action by the alkyl-lipid Ohmline. Oncotarget.

[81] Wei W.C., Huang W.C., Lin Y.P., Becker E.B., Ansorge O., Flockerzi V., Conti D., Cenacchi G., Glitsch M.D. (2017). Functional expression of calcium-permeable canonical transient receptor potential 4-containing channels promotes migration of medulloblastoma cells. J. Physiol..

[82] Chen Z., Zhu Y., Dong Y., Zhang P., Han X., Jin J., Ma X. (2017). Overexpression of TrpC5 promotes tumor metastasis via the HIF-1α–Twist signaling pathway in colon cancer. Clin. Sci..

[83] Fioro Pla A., Gkika D. (2013). Emerging role of TRP channels in cell migration: From tumor vascularization to metastasis. Front. Physiol..

[84] Fels B., Bulk E., Pethő Z., Schwab A. (2018). The role of TRP channels in the metastatic cascade. Pharmaceuticals.

[85] Wang D., Li X., Liu J., Li J., Li L.J., Qiu M.X. (2014). Effects of TRPC6 on invasibility of low-differentiated prostate cancer cells. Asian Pac. J. Trop. Med..

[86] Déliot N., Constantin B. (2015). Plasma membrane calcium channels in cancer: Alterations and consequences for cell proliferation and migration. Biochim. Biophys. Acta (BBA) Biomembr..

[87] Chinigò G., Pla A.F., Gkika D. (2020). TRP channels and small GTPases interplay in the main hallmarks of metastatic cancer. Front. Pharmacol..

[88] Oda K., Umemura M., Nakakaji R., Tanaka R., Sato I., Nagasako A., Oyamada C., Baljinnyam E., Katsumata M., Xie L.H. (2017). Transient receptor potential cation 3 channel regulates melanoma proliferation and migration. J. Physiol. Sci..

[89] Yang L.L., Liu B.C., Lu X.Y., Yan Y., Zhai Y.J., Bao Q., Doetsch P.W., Deng X., Thai T.L., Alli A.A. (2017). Inhibition of TRPC6 reduces non-small cell lung cancer cell proliferation and invasion. Oncotarget.

[90] Xu J., Yang Y., Xie R., Liu J., Nie X., An J., Wen G., Liu X., Jin H., Tuo B. (2018). The NCX1/TRPC6 complex mediates TGFβ-driven migration and invasion of human hepatocellular carcinoma cells. Cancer Res..

[91] Song Y., Liu G., Liu S., Chen R., Wang N., Liu Z., Zhang X., Xiao Z., Liu L. (2019). Helicobacter pylori upregulates TRPC6 via Wnt/β-catenin signaling to promote gastric cancer migration and invasion. OncoTargets Ther..

[92] Almasi S., Sterea A.M., Fernando W., Clements D.R., Marcato P., Hoskin D.W., Gujar S., El Hiani Y. (2019). TRPM2 ion channel promotes gastric cancer migration, invasion and tumor growth through the AKT signaling pathway. Sci. Rep..

[93] Huang B., Chang C., Wang B.L., Li H. (2019). ELK1-induced upregulation of lncRNA TRPM2-AS promotes tumor progression in gastric cancer by regulating miR-195/HMGA1 axis. J. Cell. Biochem..

[94] Hong X., Yu J.J. (2019). MicroRNA-150 suppresses epithelial-mesenchymal transition, invasion, and metastasis in prostate cancer through the TRPM4-mediated β-catenin signaling pathway. Am. J. Physiol. Cell Physiol..

[95] Maeda T., Suzuki A., Koga K., Miyamoto C., Maehata Y., Ozawa S., Hata R.I., Nagashima Y., Nabeshima K., Miyazaki K. (2017). TRPM5 mediates acidic extracellular pH signaling and TRPM5 inhibition reduces spontaneous metastasis in mouse B16-BL6 melanoma cells. Oncotarget.

[96] Liu L., Wu N., Wang Y., Zhang X., Xia B., Tang J., Cai J., Zhao Z., Liao Q., Wang J. (2019). TRPM7 promotes the epithelial–mesenchymal transition in ovarian cancer through the calcium-related PI3K/AKT oncogenic signaling. J. Exp. Clin. Cancer Res..

[97] Yee N.S., Kazi A.A., Li Q., Yang Z., Berg A., Yee R.K. (2015). Aberrant over-expression of TRPM7 ion channels in pancreatic cancer: Required for cancer cell invasion and implicated in tumor growth and metastasis. Biol. Open.

[98] Su F., Wang B.F., Zhang T., Hou X.M., Feng M.H. (2019). TRPM7 deficiency suppresses cell proliferation, migration, and invasion in human colorectal cancer via regulation of epithelial-mesenchymal transition. Cancer Biomark..

[99] Wang Y., Yang Z., Meng Z., Cao H., Zhu G., Liu T., Wang X. (2014). Knockdown of TRPM8 suppresses cancer malignancy and enhances epirubicin-induced apoptosis in human osteosarcoma cells. Int. J. Biol. Sci..

[100] Liu J., Chen Y., Shuai S., Ding D., Li R., Luo R. (2014). TRPM8 promotes aggressiveness of breast cancer cells by regulating EMT via activating AKT/GSK-3β pathway. Tumor Biol..

[101] Wang G., Cao R., Qian K., Peng T., Yuan L., Chen L., Cheng S., Xiong Y., Ju L., Wang X. (2020). TRPM8 Inhibition Regulates the Proliferation, Migration and ROS Metabolism of Bladder Cancer Cells. OncoTargets Ther..

[102] Winchester W.J., Gore K., Glatt S., Petit W., Gardiner J.C., Conlon K., Postlethwaite M., Saintot P.P., Roberts S., Gosset J.R. (2014). Inhibition of TRPM8 channels reduces pain in the cold pressor test in humans. J. Pharmacol. Exp. Ther..

[103] Xu S., Zhang L., Cheng X., Yu H., Bao J., Lu R. (2018). Capsaicin inhibits the metastasis of human papillary thyroid carcinoma BCPAP cells through the modulation of the TRPV1 channel. Food Funct..

[104] Monet M., Lehen’kyi V.Y., Gackiere F., Firlej V., Vandenberghe M., Roudbaraki M., Gkika D., Pourtier A., Bidaux G., Slomianny C. (2010). Role of cationic channel TRPV2 in promoting prostate cancer migration and progression to androgen resistance. Cancer Res..

[105] Oulidi A., Bokhobza A., Gkika D., Abeele F.V., Lehen’kyi V.Y., Ouafik L.H., Mauroy B., Prevarskaya N. (2013). TRPV2 mediates adrenomedullin stimulation of prostate and urothelial cancer cell adhesion, migration and invasion. PLoS ONE.

[106] Cappelli H.C., Kanugula A.K., Adapala R.K., Amin V., Sharma P., Midha P., Paruchuri S., Thodeti C.K. (2019). Mechanosensitive TRPV4 channels stabilize VE-cadherin junctions to regulate tumor vascular integrity and metastasis. Cancer Lett..

[107] Lee W.H., Choong L.Y., Jin T.H., Mon N.N., Chong S., Liew C.S., Putti T., Lu S.Y., Harteneck C., Lim Y.P. (2017). TRPV4 plays a role in breast Cancer cell migration via Ca^2+^-dependent activation of AKT and downregulation of E-cadherin cell cortex protein. Oncogenesis.

[108] Wang H., Zhang B., Wang X., Mao J., Li W., Sun Y., Yuan Y., Ben Q., Hua L., Qian A. (2020). TRPV4 Overexpression Promotes Metastasis Through Epithelial–Mesenchymal Transition in Gastric Cancer and Correlates with Poor Prognosis. OncoTargets Ther..

[109] Li X., Cheng Y., Wang Z., Zhou J., Jia Y., He X., Zhao L., Dong Y., Fan Y., Yang X. (2020). Calcium and TRPV4 promote metastasis by regulating cytoskeleton through the RhoA/ROCK1 pathway in endometrial cancer. Cell Death Dis..

[110] Ren T., Zhang H., Wang J., Zhu J., Jin M., Wu Y., Guo X., Ji L., Huang Q., Yang H. (2017). MCU-dependent mitochondrial Ca^2+^ inhibits NAD+/SIRT3/SOD2 pathway to promote ROS production and metastasis of HCC cells. Oncogene.

[111] Jin M., Wang J., Ji X., Cao H., Zhu J., Chen Y., Yang J., Zhao Z., Ren T., Xing J. (2019). MCUR1 facilitates epithelial-mesenchymal transition and metastasis via the mitochondrial calcium dependent ROS/Nrf2/Notch pathway in hepatocellular carcinoma. J. Exp. Clin. Cancer Res..

[112] Tang S., Wang X., Shen Q., Yang X., Yu C., Cai C., Cai G., Meng X., Zou F. (2015). Mitochondrial Ca^2+^ uniporter is critical for store-operated Ca^2+^ entry-dependent breast cancer cell migration. Biochem. Biophys. Res. Commun..

[113] Tosatto A., Sommaggio R., Kummerow C., Bentham R.B., Blacker T.S., Berecz T., Duchen M.R., Rosato A., Bogeski I., Szabadkai G. (2016). The mitochondrial calcium uniporter regulates breast cancer progression via HIF-1α. EMBO Mol. Med..

[114] Zheng X., Lu S., He Z., Huang H., Yao Z., Miao Y., Cai C., Zou F. (2020). MCU-dependent negative sorting of miR-4488 to extracellular vesicles enhances angiogenesis and promotes breast cancer metastatic colonization. Oncogene.

[115] Wang X., Song X., Cheng G., Zhang J., Dong L., Bai J., Luo D., Xiong Y., Li S., Liu F. (2020). The Regulatory Mechanism and Biological Significance of Mitochondrial Calcium Uniporter in the Migration, Invasion, Angiogenesis, and Growth of Gastric Cancer. OncoTargets Ther..

[116] Kanwar N., Carmine-Simmen K., Nair R., Wang C., Moghadas-Jafari S., Blaser H., Tran-Thanh D., Wang D., Wang P., Wang J. (2020). Amplification of a calcium channel subunit CACNG4 increases breast cancer metastasis. Ebiomedicine.

[117] Marques R., Peres C.G., Vaz C.V., Gomes I.M., Figueira M.I., Cairrão E., Verde I., Maia C.J., Socorro S. (2015). 5α-Dihydrotestosterone regulates the expression of L-type calcium channels and calcium-binding protein regucalcin in human breast cancer cells with suppression of cell growth. Med. Oncol..

[118] Maiques O., Barceló C., Panosa A., Pijuan J., Orgaz J.L., Rodriguez-Hernandez I., Matas-Nadal C., Tell G., Vilella R., Fabra A. (2018). T-type calcium channels drive migration/invasion in BRAFV 600E melanoma cells through Snail1. Pigment Cell Melanoma Res..

[119] Sharma S., Wu S.Y., Jimenez H., Xing F., Zhu D., Liu Y., Wu K., Tyagi A., Zhao D., Lo H.W. (2019). Ca^2+^ and CACNA1H mediate targeted suppression of breast cancer brain metastasis by AM RF EMF. EBioMedicine.

[120] Grasset E.M., Bertero T., Bozec A., Friard J., Bourget I., Pisano S., Lecacheur M., Maiel M., Bailleux C., Emelyanov A. (2018). Matrix stiffening and EGFR cooperate to promote the collective invasion of cancer cells. Cancer Res..

[121] Phiwchai I., Thongtem T., Thongtem S., Pilapong C. (2020). Liver Cancer Cells Uptake Labile Iron via L-type Calcium Channel to Facilitate the Cancer Cell Proliferation. Cell Biochem. Biophys..

[122] Ono Y., Sorimachi H. (2012). Calpains—An elaborate proteolytic system. Biochim. Biophys. Acta (BBA) Proteins Proteom..

[123] Chen J., Wu Y., Zhang L., Fang X., Hu X. (2019). Evidence for calpains in cancer metastasis. J. Cell. Physiol..

[124] Xu C., Yu X., Zhu Y., Cai Z., Yu L., Lin Y., Yu H., Xue Z., Zhou L. (2019). Overexpression of calpain-1 predicts poor outcome in patients with colorectal cancer and promotes tumor cell progression associated with downregulation of FLNA. Oncol. Rep..

[125] Yu L.M., Zhu Y.S., Xu C.Z., Zhou L.L., Xue Z.X., Cai Z.Z. (2019). High calpain-1 expression predicts a poor clinical outcome and contributes to tumor progression in pancreatic cancer patients. Clin. Transl. Oncol..

[126] Luo W., Ren Z., Gao S., Jin H., Zhang G., Zhou L., Zheng S. (2016). Clinical correlation of calpain-1 and glypican-3 expression with gallbladder carcinoma. Oncol. Lett..

[127] Mamoune A., Luo J.H., Lauffenburger D.A., Wells A. (2003). Calpain-2 as a target for limiting prostate cancer invasion. Cancer Res..

[128] Gao X., Mao Y.H., Xiao C., Li K., Liu W., Li L.Y., Pang J. (2018). Calpain-2 triggers prostate cancer metastasis via enhancing CRMP4 promoter methylation through NF-κB/DNMT1 signaling pathway. Prostate.

[129] Peng P., Wu W., Zhao J., Song S., Wang X., Jia D., Shao M., Zhang M., Li L., Wang L. (2016). Decreased expression of Calpain-9 predicts unfavorable prognosis in patients with gastric cancer. Sci. Rep..

[130] Zheng P., Chen X., Xie J., Chen X., Lin S., Ye L., Chen L., Lin J., Yu X., Zheng M. (2020). Capn4 is induced by and required for Epstein-Barr virus latent membrane protein 1 promotion of nasopharyngeal carcinoma metastasis through ERK/AP-1 signaling. Cancer Sci..

[131] Zhao C., Yuan G., Jiang Y., Xu J., Ye L., Zhan W., Wang J. (2020). Capn4 contributes to tumor invasion and metastasis in gastric cancer via activation of the Wnt/β-catenin/MMP9 signalling pathways. Exp. Cell Res..

[132] Peng P., Min L., Song S., Zhao J., Li L., Yang C., Shao M., Zhang M., Wu H., Zhang J. (2016). Elevated expression of calpain-4 predicts poor prognosis in patients with gastric cancer after gastrectomy. Int. J. Mol. Sci..

[133] Chen Y., Wang G., Wang Y., Gao X., Wang K., Li J., Xue F. (2019). Capn4 regulates migration and invasion of ovarian carcinoma cells via targeting osteopontin-mediated PI3K/AKT signaling pathway. Oncol. Lett..

[134] Li Y., Zhang Z., Zhou X., Li L., Liu Q., Wang Z., Bai X., Zhao Y., Shi H., Zhang X. (2014). The oncoprotein HBXIP enhances migration of breast cancer cells through increasing filopodia formation involving MEKK2/ERK1/2/Capn4 signaling. Cancer Lett..

[135] Cai J.J., Qi Z.X., Chen L.C., Yao Y., Gong Y., Mao Y. (2015). miR-124 suppresses the migration and invasion of glioma cells in vitro via Capn4. Oncol. Rep..

[136] Villalobo A., Berchtold M.W. (2020). The role of calmodulin in tumor cell migration, invasiveness, and metastasis. Int. J. Mol. Sci..

[137] Liu Z., Han G., Cao Y., Wang Y., Gong H. (2014). Calcium/calmodulin-dependent protein kinase II enhances metastasis of human gastric cancer by upregulating nuclear factor-κB and Akt-mediated matrix metalloproteinase-9 production. Mol. Med. Rep..

[138] Chen W., An P., Quan X.J., Zhang J., Zhou Z.Y., Zou L.P., Luo H.S. (2017). Ca^2+^/calmodulin-dependent protein kinase II regulates colon cancer proliferation and migration via ERK1/2 and p38 pathways. World J. Gastroenterol..

[139] Yu G., Cheng C.J., Lin S.C., Lee Y.C., Frigo D.E., Yu-Lee L.Y., Gallick G.E., Titus M.A., Nutt L.K., Lin S.H. (2018). Organelle-derived acetyl-CoA promotes prostate cancer cell survival, migration, and metastasis via activation of calmodulin kinase II. Cancer Res..

[140] Alessandrini F., Pezzè L., Ciribilli Y. (2017). LAMPs: Shedding light on cancer biology. Semin. Oncol..

[141] Eskelinen E.L. (2006). Roles of LAMP-1 and LAMP-2 in lysosome biogenesis and autophagy. Mol. Asp. Med..

[142] Dower C.M., Wills C.A., Frisch S.M., Wang H.G. (2018). Mechanisms and context underlying the role of autophagy in cancer metastasis. Autophagy.

[143] Dang Q., Zhou H., Qian J., Yang L., Huang J., Zhang Y., Shi W. (2018). LAMP1 Overexpression Predicts for Poor Prognosis in Diffuse Large B-cell Lymphoma. Clin. Lymphoma Myeloma Leuk..

[144] Xu Y., Cao X., Zhang S., Zhang Y., Shen Z. (2017). High expression of LAMP1 as a prognostic marker in patients with epithelial ovarian cancer. Int. J. Clin. Exp. Pathol..

[145] Wang Q., Yao J., Jin Q., Wang X., Zhu H., Huang F., Wang W., Qiang J., Ni Q. (2017). LAMP1 expression is associated with poor prognosis in breast cancer. Oncol. Lett..

[146] Lu M., Zhu H., Wang X., Zhang D., Xiong L., Zhu J., Mao Y., Qiang J. (2016). LAMP1 expression is associated with malignant behaviours and predicts unfavourable prognosis in laryngeal squamous cell carcinoma. Pathology.

[147] Chen H., Li L., Hu J., Zhao Z., Ji L., Cheng C., Zhang G., Li Y., Chen H., Pan S. (2019). UBL4A inhibits autophagy-mediated proliferation and metastasis of pancreatic ductal adenocarcinoma via targeting LAMP1. J. Exp. Clin. Cancer Res..

[148] Li L., Wang W., Zhang R., Liu J., Yu J., Wu X., Xu Y., Ma M., Huang J. (2017). High expression of LAMP2 predicts poor prognosis in patients with esophageal squamous cell carcinoma. Cancer Biomark..

[149] Liao X., Chen Y., Liu D., Li F., Li X., Jia W. (2015). High expression of LAMP3 is a novel biomarker of poor prognosis in patients with esophageal squamous cell carcinoma. Int. J. Mol. Sci..

[150] Wang D., Cao X., Zhang Y., Liu Y., Yao C., Ge W., Xu Y. (2017). LAMP3 expression correlated with poor clinical outcome in human ovarian cancer. Tumor Biol..

[151] Gui Y., Liu W.B., Chen H., Ma J.L., Li J.S. (2018). Expression of LAMP3 and its correlation with clinicopathologic characteristics and prognosis in hepatocellular carcinoma. Int. J. Clin. Exp. Pathol..

[152] Huang F., Ma G., Zhou X., Zhu X., Yu X., Ding F., Cao X., Liu Z. (2020). Depletion of LAMP3 enhances PKA-mediated VASP phosphorylation to suppress invasion and metastasis in esophageal squamous cell carcinoma. Cancer Lett..

[153] Staresinic B., Jesenko T., Kamensek U., Frandsen S.K., Sersa G., Gehl J., Cemazar M. (2018). Effect of calcium electroporation on tumour vasculature. Sci. Rep..

[154] Mahalingam D., Wilding G., Denmeade S., Sarantopoulas J., Cosgrove D., Cetnar J., Azad N., Bruce J., Kurman M., Allgood V.E. (2016). Mipsagargin, a novel thapsigargin-based PSMA-activated prodrug: Results of a first-in-man phase I clinical trial in patients with refractory, advanced or metastatic solid tumours. Br. J. Cancer.

[155] Holdhoff M., Ye X., Supko J.G., Nabors L.B., Desai A.S., Walbert T., Lesser G.J., Read W.L., Lieberman F.S., Lodge M.A. (2017). Timed sequential therapy of the selective T-type calcium channel blocker mibefradil and temozolomide in patients with recurrent high-grade gliomas. Neuro Oncol..

